# OX40/OX40L axis: not a friend in autoimmunity

**DOI:** 10.18632/oncotarget.4973

**Published:** 2015-07-21

**Authors:** Hideki Ueno, Patrick Blanco

**Affiliations:** UMR-CNRS 5164, CIRID, Bordeaux cedex, France

**Keywords:** Immunology and Microbiology Section, Immune response, Immunity, autoimmunity, costimulatory molecule, follicular helper T cell

Systemic Lupus Erythematosus (SLE) is a chronic autoimmune disease characterized by a loss of tolerance toward nuclear components, and multiple organs such as kidney, brain, vessels or skin are affected. SLE presents a waxing and waning course, rendering its outcome hardly predictable. A better understanding of human SLE pathogenesis is direly needed because only few effective treatments are available. While the precise immunological events that trigger the onset of SLE remain unknown, chronic activation of the dendritic cell (DC) system plays an important role for the activation of autoreactive T and B lymphocytes while overwhelming natural regulatory mechanisms. Generation of autoantibodies targeting broad repertoire of self antigens and formation of immune complexes are the hallmark of SLE. Central to antibody production is the interactions between CD4^+^ T cells and B cells particularly in germinal centers (GCs), the site of affinity maturation and the subsequent generation of memory B cells and long-lived plasma cells. In SLE, a majority of IgG class autoantibody-producing B cells are somatically mutated indicating that they are derived from GCs [1]. Consistently, recent data in humans and mice show that overrepresentation of T follicular helper cells (Tfh), a CD4^+^ T cell subset specialized in helping B cells in GCs, is associated with autoimmunity including SLE [2]. However, the mechanism that leads to the exaggerated Tfh response in SLE was largely unknown. Our recent study demonstrated that the OX40-OX40 ligand (OX40L) axis contributes to the lupus pathogenesis in this context [3].

OX40-OX40L belongs to the TNFR-TNF superfamily members. OX40 expressed by activated T cells delivers costimulatory signals required for their optimal proliferation and survival [4]. A number of previous mouse studies demonstrated the pathogenic role of the OX40-OX40L axis in autoimmune diseases, and disruption of this axis was shown to be beneficial for the prevention and the treatment of the diseases [4]. However, whether the OX40-OX40L axis indeed plays pathogenic roles in human SLE was unclear. We found that OX40L was overexpressed by myeloid antigen presenting cells (APCs) in blood and in inflamed tissues in adult and pediatric SLE patients [3]. The frequency of circulating OX40L-expressing myeloid APCs positively correlated with disease activity assessed by the SLE Disease Activity Index. Importantly, our study shows that OX40 signal promotes human naive and memory CD4^+^ T cells to become functional B cell helpers that share phenotype and the gene profiles with Tfh cells. Furthermore, the frequency of activated blood Tfh cells correlated with the frequency of circulating OX40L^+^ myeloid APCs in SLE. Collectively, these observations suggest that activated OX40L^+^ myeloid-APCs represent a key player for the generation and/or the activation of Tfh cells in SLE.

Then what triggers OX40L expression by myeloid APCs in SLE? We previously demonstrated that stimulation with serum obtained from active SLE patients renders healthy monocytes to become cells with features of DCs. Such property of SLE sera was mainly mediated by type I interferon [5]. To our surprise, while stimulation with SLE sera induced monocytes to express OX40L, type I interferon was not involved in OX40L expression. Instead, we found that RNA-containing immune complexes present in SLE sera induced OX40L expression by monocytes [3]. OX40L expression was totally dependent on TLR7, as monocytes stimulated with SLE sera in the presence of specific TLR7 inhibitor did not express OX40L. Of note, while monocytes do not express much TLR7, the stimulation with SLE sera rapidly upregulated TLR7 expression (unpublished observations).

The pathogenic roles of immune complexes containing self nucleic acid are well established in SLE. While many of the identified mechanisms involve the activation of the innate immune system and consequent inflammation, our study shows that immune complexes also activate the adaptive immune system. The immune complexes containing RNA induce OX40L expression by monocytes and macrophages via TLR7. These OX40L^+^ myeloid APCs promote Tfh responses, which further accelerate the generation of autoantibodies including those against self nucleic acid. Thus, the OX40-OX40L axis likely provides an amplification loop of the generation of autoantibodies in SLE.

It is noteworthy that myeloid APCs were not the only cells expressing OX40L in inflammatory tissues in SLE. OX40L can be expressed by a broad range of immune cells including B cells, vascular endothelial cells, mast cells, activated NK cells, and activated CD4^+^ T cells [4], and which cells are expressing OX40L in inflammatory tissues remains to be established. It is possible that these cells may also act as additional APCs. Alternatively, these cells may shed OX40L in a soluble form which can deliver activation signals via OX40. Nonetheless, our study provides a new line of evidence that TLR7 and the OX40-OX40L can be potentially therapeutic targets in SLE.
